# Exoskeleton-assisted walking improves pulmonary function and walking parameters among individuals with spinal cord injury: a randomized controlled pilot study

**DOI:** 10.1186/s12984-021-00880-w

**Published:** 2021-05-24

**Authors:** Xiao-Na Xiang, Hui-Yan Zong, Yi Ou, Xi Yu, Hong Cheng, Chun-Ping Du, Hong-Chen He

**Affiliations:** 1 Department of Rehabilitation Medicine, West China Hospital, Sichuan University, Chengdu, 610041 Sichuan People’s Republic of China; 2 School of Rehabilitation Sciences, West China School of Medicine, Sichuan University, Chengdu, 610041 Sichuan People’s Republic of China; 3 Key Laboratory of Rehabilitation Medicine in Sichuan Province, West China Hospital, Sichuan University, Chengdu, 610041 Sichuan People’s Republic of China; 4 University of Electronic Science and Technology of China, Chengdu, 611731 Sichuan People’s Republic of China

**Keywords:** Spinal cord, Pulmonary, Exercise, Exoskeleton, 6MWT, Rehabilitation

## Abstract

**Background:**

Exoskeleton-assisted walking (EAW) is expected to improve the gait of spinal cord injury (SCI) individuals. However, few studies reported the changes of pulmonary function (PF) parameters after EAW trainings. Hence, we aimed to explore the effect of EAW on PF parameters, 6-min walk test (6MWT) and lower extremity motor score (LEMS) in individuals with SCI and to compare those with conventional trainings.

**Methods:**

In this prospective, single-center, single-blinded randomized controlled pilot study, 18 SCI participants were randomized into the EAW group (n = 9) and conventional group (n = 9) and received 16 sessions of 50–60 min training (4 days/week, 4 weeks). Pulmonary function parameters consisting of the forced vital capacity (FVC), forced expiratory volume in 1 s (FEV_1_), forced expiratory flow (FEF), peak expiratory flow, and maximal voluntary ventilation, 6MWT with assisted devices and LEMS were reported pre- and post-training.

**Results:**

Values of FVC (p = 0.041), predicted FVC% (p = 0.012) and FEV_1_ (p = 0.013) were significantly greater in EAW group (FVC: 3.8 ± 1.1 L; FVC% _pred_ = 94.1 ± 24.5%; FEV_1_: 3.5 ± 1.0 L) compared with conventional group (FVC: 2.8 ± 0.8 L; FVC% _pred_ = 65.4 ± 17.6%; FEV_1_: 2.4 ± 0.6 L) after training. Participants in EAW group completed 6MWT with median 17.3 m while wearing the exoskeleton. There was no difference in LEMS and no adverse event.

**Conclusions:**

The current results suggest that EAW has potential benefits to facilitate PF parameters among individuals with lower thoracic neurological level of SCI compared with conventional trainings. Additionally, robotic exoskeleton helped walking. *Trial registration*: Registered on 22 May 2020 at Chinese Clinical Trial Registry (ChiCTR2000033166). http://www.chictr.org.cn/edit.aspx?pid=53920&htm=4.

**Supplementary Information:**

The online version contains supplementary material available at 10.1186/s12984-021-00880-w.

## Background

Spinal cord injury (SCI) is a worldwide life-disrupting pathological condition with estimated 17,810 injuries occurred in the United States in 2017 [1, 2], and 3.5 per million in the United Kingdom each year [3]. Respiratory complications are common after SCI that account for 5.4% of the causes of death [4] and have a great impact on reducing quality of life and life expectancy. On the other hand, pulmonary capacity may have implications for exercise performance because oxygen is essential for organ system metabolism [5]. Hence, a good pulmonary function (PF) is of vital importance for individuals with SCI.

Pulmonary function parameters are affected by the paralyzed degree (complete or partial) of the respiratory muscles [6] which consist of diaphragm, intercostal muscles, accessory respiratory muscles, and abdominal muscles. Moreover, PF parameters are associated with the level of damaged spinal cord [7, 8]. Therefore, doctors and therapists concerned more about the PF among higher injury level individuals. Specific trainings, such as respiratory muscle training [9] and abdominal drawing-in maneuver [10] are frequently used for them. Unfortunately, SCI individuals with lower injury level also complained about the bad results of pulmonary function test (PFT) [11]. Walking and running are key elements of an aerobic exercise program. Additionally, they are useful to keep pulmonary and exercise capacity [12, 13]. However, the paralysis of lower limb and osteoporosis after SCI would make individuals difficult to walk or run. Instead, upper limb aerobic exercise [14], strength training [15], and balance training [16–18] are common exercises to maintain exercise capacity for these individuals. Nonetheless, previous study reported that these were hard to improve the resting lung function or exercise performance [19]. Hence, individuals assessed as lower extremity motor complete lesions need assisted devices to complete walking and that may result in improvement in the pulmonary and exercise capacity.

Recently, exoskeleton-assisted walking (EAW) has been confirmed to help individuals with thoracic and lumbar SCI to walk safely [20–22]. Despite the potential walking benefits of EAW, there are few studies manifested that used EAW trainings for PF improving. A quantity of studies has manifested the improvements of metabolic responses, such as heart rate and VO_2 max_ [23–25] during the training program. Nevertheless, none of them has focused on the changes of PF parameters by PFT which contained the vital capacity, forced vital capacity (FVC), forced expiratory volume in 1 second (FEV_1_), forced expiratory flow (FEF_25/50/75_), peak expiratory flow (PEF), and maximal voluntary ventilation (MVV). Besides this, previous trials were non-randomized controlled trials. Therefore, this randomized controlled study primarily aimed at finding out whether the EAW trainings are different from conventional rehabilitation trainings in improving PF parameters among SCI individuals. Our hypotheses were that EAW training can improve the PF parameters and maintain the lower extremity motor score (LEMS) which does not make a difference relative to that made by conventional training. Second, individuals could complete walking while wearing the exoskeleton. To our knowledge, this is the first clinical trial to concern about the PF parameters by EAW training.

## Methods

### Study design and ethics statement

This was a single-blinded, randomized controlled efficacy trial with 2 parallel groups and intention-to-treat analysis. The study protocol has been registered at Chinese Clinical Trial Registry (ChiCTR2000033166) and approved by the medical ethics committee of West China Hospital of Sichuan University (#19-667). All patients were informed of the procedure, the use of their data and images for research. They understood the purposes and provided written informed consent according to the 1964 Declaration of Helsinki prior to their participation.

### Participant recruitment

From May 2020 to August 2020, we prospectively enrolled all adult individuals with a diagnosed SCI below T3 and above L2 at least 1 month. Participants were recruited from inpatients in 3 units of Rehabilitation center, West China Hospital, Sichuan University. None of them had EAW training experience before. In addition, the eligible individuals met the following inclusion criteria: (1) American spinal injuries association impairment scale [26] (ASIA) classified with A, B or C, (2) the height was between 1.50 m and 1.85 m and (3) stopped smoking for over 6 months. Individuals were excluded if: (1) spasticity of any the lower extremity muscle scored over 2 according to the Modified Ashworth Scale [27], (2) with unstable fracture, (3) diagnosed with severe osteoporosis (bone mineral density t-score < − 3.5), (4) with any respiratory or other neurological diseases.

### Randomization and blinding

The individuals were randomly divided into the EAW group or conventional group in a 1:1 ratio by simple randomization method, using computer-generated simple random tables. The sequences were preserved using closed envelop method by one researcher who did not participant in the trainings and assessments (H.C.H). PFT and LEMS assessment of individuals pre- and post-training were performed by the same two clinical researchers (X.Y. and Y.O) who were blinded and did not know whether the individual was in EAW or conventional group. 6MWT was performed by two researchers who participated in EAW training (H.C and C.P.D) and conventional training (H.Y.Z). Clinical data was recorded after averaging.

### Interventions

**Fig. 1:**
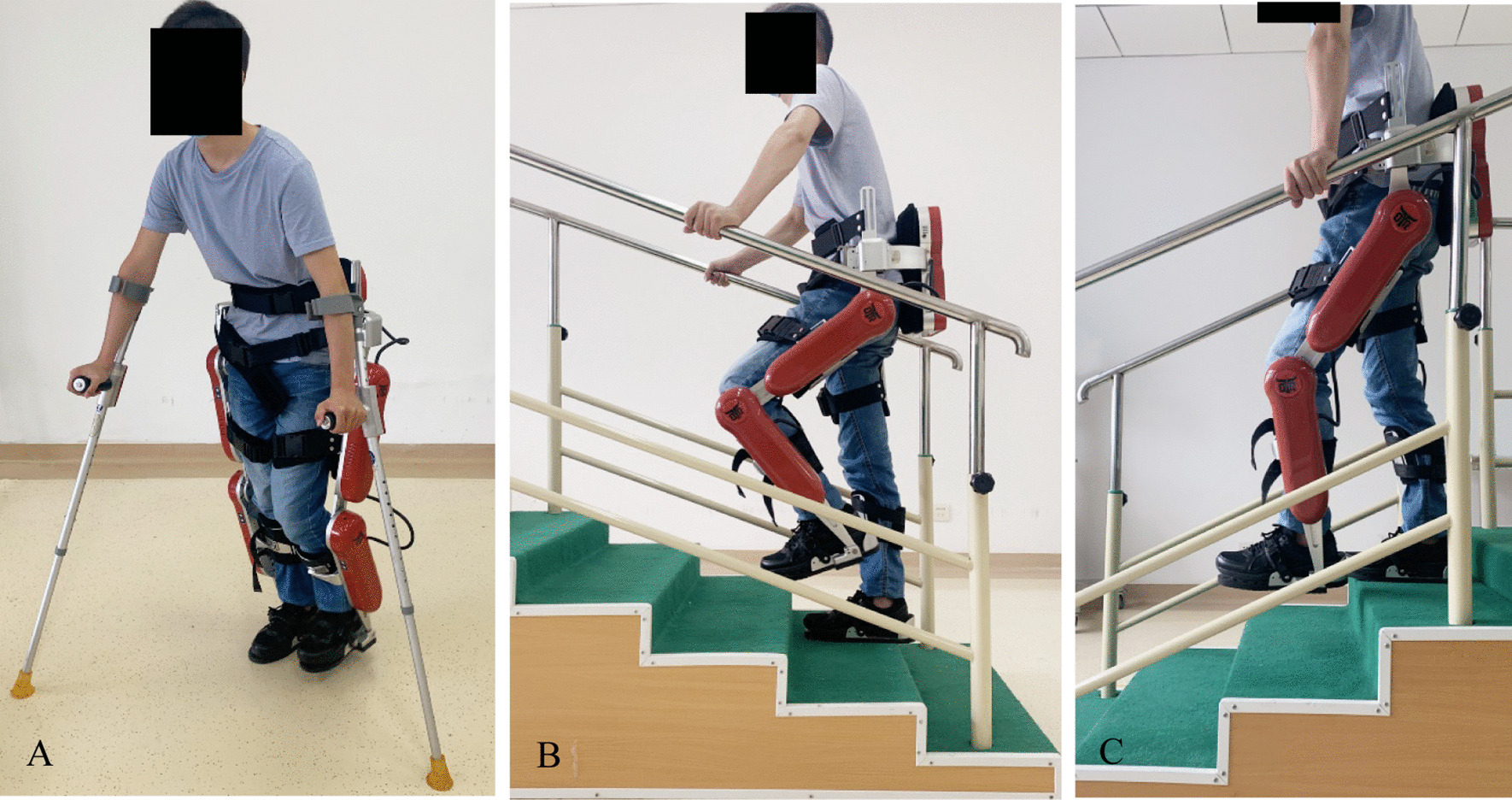
AIDER powered exoskeleton illustration used in this study. **A** Walk in exoskeleton; **B** go upstairs in exoskeleton; **C** go downstairs in exoskeleton

The AIDER (AssItive DEvice for paRalyzed patient) powered robotic exoskeleton (generation IV, Buffalo Robot Technology Co. Ltd, Chengdu, China) was used for the EAW training. All subjects were individually fitted to the robotic exoskeleton according to pelvic width, thigh length, and shank length. Exoskeleton-assisted walking training program was conducted consecutive 4 days a week, 16 training sessions in total. Every training session lasted 50–60 min containing interval rest as needed by standing, leaning on the wall, or sitting while wearing the device. Training session included sitting, standing, walking, climbing stairs and slope with maximal assistance-walking mode (Fig. 1) and reaching 40–60% maximal heart rate (HR, HR_max_ = 220—age)[28] that is checked with the values of a heart rate sensor (Polar H10, POLAR^®^ China).

For the conventional group, the rehabilitation program included strength training using dumbbell between 5 and 20 kg, aerobic exercise, such as walking training with brace as well as static and dynamic balance training in sitting or standing position. The conventional rehabilitation training had the same intensity, duration, and frequency as EAW training (40–60% HR_max_, 50–60 min/session, 4 days/week, 4 weeks). Medications and rehabilitation nursing were ordered based on the medical condition.

### Measures

Outcome measures were collected and analyzed at the baseline and end of 16-session intervention period.

#### Primary outcome

Pulmonary function test was completed with a computerized spirometer (Vyntus™ SPIRO PC Spirometer, Vyaire Medical Inc., Mettawa, US) based onthe standardized procedures as the American Thoracic Society [29] described. To determine PF parameters, participants performed PFT seating in the wheelchair and were forbidden to disclose their intervention assignment to the assessor. The PFT was performed with the participants wearing a nose clip. If the participant coughed or made a mistake, the numerical values were not recorded. Three repeated maneuvers were performed, separated by a five-minute rest and the best result was recorded automatically. The PFT consisted of the assessments of FVC, FEV_1_, FEF_25/50/75_, PEF, and MVV.

Forced vital capacity refers to the total capacity of air that can be blown out by maxima forced expiration following maximal inspiration. Forced expiratory volume in 1 s refers to the capacity of air that is blown out for a single second. Force expiratory flow_25/50/75_ means forced expiratory flow at 25, 50 and 75% of the FVC. Peak expiratory flow reflects the intensity of respiratory muscles. Maximal voluntary ventilation refers to the maximum volume of air, a subject can breathe over a specified period [30].

#### Secondary outcomes

The 6MWT (6-min walk test) was performed in door that is aimed to determine walking ability. Additionally, it can manifest cardiorespiratory endurance through evaluating the walking distance in accordance with the guidelines of the American Thoracic Society [31]. At the beginning and end of the test, clinical researchers recorded the participant’s HR, and peripheral oxygen saturation (SpO_2_). In additional, the level of effort at the end of the test was reported by the rate of perceived exertion (RPE) based on the Borg scale [32]. Participants ambulated using their preferred stability aid (either crutches or walker). Individuals in EAW group were allowed to wear the exoskeleton, while those in conventional group using the knee-ankle–foot orthoses if they had one. The outcome of distance will be recorded as 0 and others be recorded according to the facts, if the individuals cannot walk. Moreover, lower extremity motor score [33] and ASIA scores were reported to demonstrate the recovery of muscle strength and neurological level.

### Data analysis

The statisticians (X.N.X and J.D) were blinded to the program and completed analyses utilized SPSS version 25 (SPSS Inc, Chicago, Illinois). The Shapiro–Wilk test was used to determine if data were normally distributed. These were recorded as means ± standard deviations (SDs), others were described as median and inter quartile range (IQR) where necessary. The mean values (Δ) of post-intervention minus pre-intervention were recorded. Independent Student t-test was used to compare continuous data related to clinical features between two groups. Paired Test Student t-test was used to compare varies between pre- and post-intervention. Furthermore, the Fisher’s exact and Pearson’s Chi-square tests were used if the data were categorical variables. Wilcoxon rank-sum test and Mann–Whitney U test were used if the data was not normally distributed. Pearson correlation test was performed to discuss the relation between the distance of 6MWT and changes of PF parameters. In all statistical tests, p < 0.05 was defined as significant.

## Results

### Participants

**Fig. 2:**
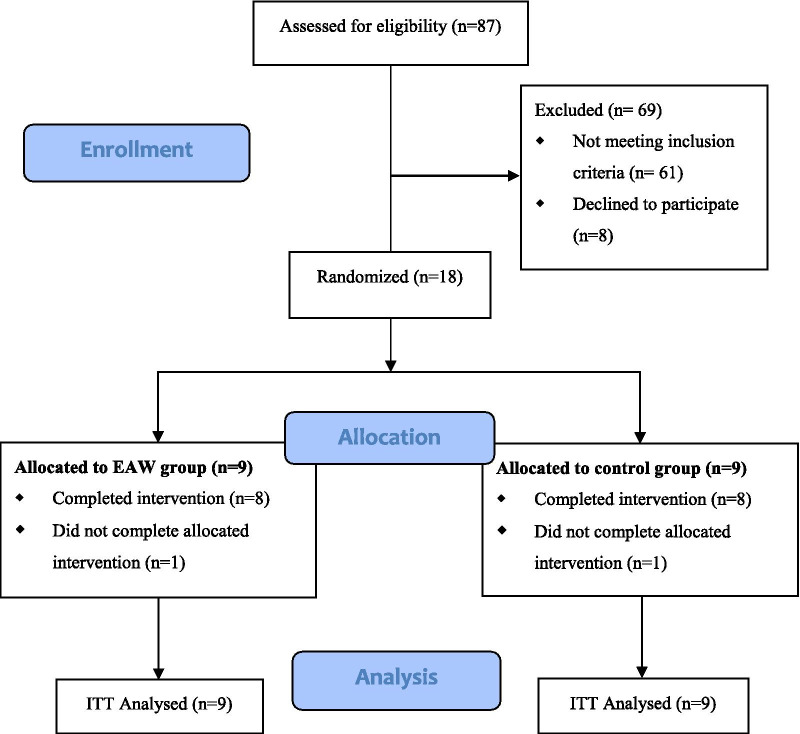
CONSORT diagram of enrollment of participants into the study

A total of 87 individuals with SCI were screened enrollment, of which 69 were excluded as per exclusion criteria (n = 61) or declining to commit the full participation (n = 8). Eighteen eligible individuals were randomized to either EAW group (n = 9) or conventional group (n = 9). The Consolidated Standards of Reporting Trials (CONSORT) diagram is showed in Fig. 2. Of the 9 individuals randomized to the EAW group, 8 have completed the EAW intervention training sessions but one individual refused to continue participating after experiencing severe anxiety episodes in the robotic exoskeleton. In the conventional group, one individual did not accept the final assessment because of own desire to be discharged from hospital. Two groups were comparable on the baseline characteristics (Table 1). At the end point of the final training session, rehabilitation evaluation and assessments were performed and no change in level and classification of neurological injury were detected.

**Table 1:** Baseline characteristics by intervention group

Characteristic	EAW group (n = 9)	Conventional group (n = 9)	p-value
Demographic			
Age, mean ± SD, year	39.8 ± 12.2	36.6 ± 11.8	0.577
Sex, No. (%)			
Women	7 (77.8)	8 (88.9)	
Men	2 (22.2)	1 (11.1)	
Clinical			
Diagnosis, No. (%)			
TSCI	7 (77.8)	7 (77.8)	
Myelitis	2 (22.2)	2 (22.2)	
LOI, No. (%)			
T4–T10	5 (55.6)	4 (44.4)	
T11 and below	4 (44.4)	5 (55.6)	
AIS, No. (%)			
A	8 (88.9)	4 (44.4)	
B	0	2 (22.2)	
C	1 (11.1)	3 (33.3)	
DOI, median (IQR), month	2.0 (4.5)	2.0 (0.5)	0.340
MAS, No. (%)			
0	9 (100)	8 (88.9)	
1	0	1 (11.1)	

*EAW* exoskeleton-assisted walking, *TSCI* traumatic spinal cord injury, *LOI* level of injury, *AIS* international standards for the neurological classification of an SCI, *DOI* duration of injury

### Adverse events

There were no adverse events related to treatment in either group.

### Primary outcome

**Fig. 3:**
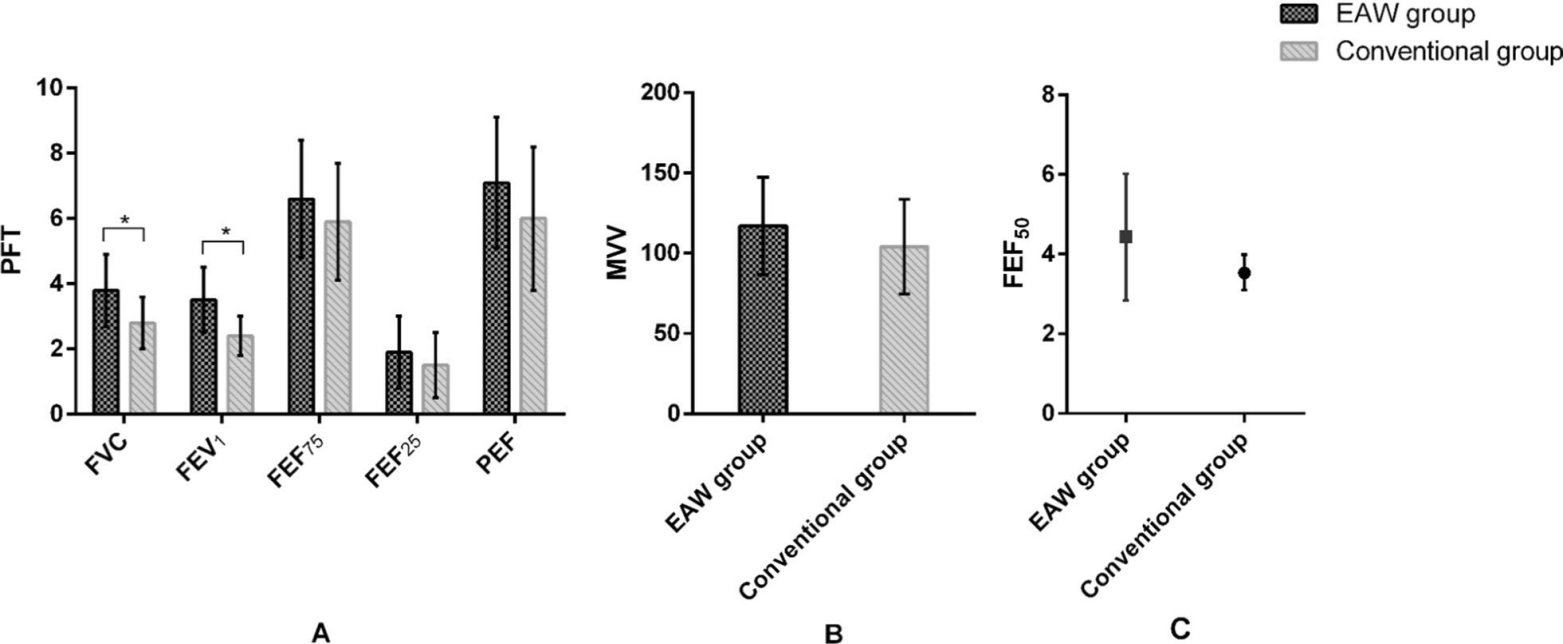
Comparisons of the improvements in pulmonary function test (PFT) between two groups. **A** Results of FVC, FEV_1_, FEF_75_, FEF_25_, and PEF; **B** result of MVV; **C** result of FEF_50_. *PFT* pulmonary function test, *p < 0.05, *EAW* exoskeleton-assisted walking group, *FVC* forced vital capacity, *FEV*_*1*_ forced expiratory volume in 1 s, *FEF* forced expiratory flow, *PEF* peak expiratory flow, *MVV* maximal voluntary ventilation

The primary outcome was measured based on PFT. Detail numerical results of PFT are provided in Table 2. The results of FVC (t = 2.224; p = 0.041) and predicted FVC% (t = 2.848, p = 0.012) showed significant differences between EAW group and conventional group. Moreover, there was statistically significant difference in FEV_1_ (t = 2.779; p = 0.013) between groups. Nevertheless, there were no statistically significant differences in FEF_75_ (t = 0.803; p = 0.434), FEF_50_ (Z = 0.927; p = 0.354), FEF_25_ (t = 0.834; p = 0.417), PEF (t = 1.097; p = 0.289), and MVV (t = 0.935; p = 0.364) (Fig. 3).

**Table 2:** Outcomes of pulmonary function test (PFT) in accordance with the training methods

Characteristic, mean ± SD	EAW group (n = 9)			Conventional group (n = 9)			p-values between two groups	
	Pre	Post	p-value in group	Pre	Post	p-value in group	Pre	Post
FVC, L	3.1 ± 1.1	3.8 ± 1.1	0.052	3.0 ± 0.9	2.8 ± 0.8	0.285	0.800	0.041
FVC% _pred_	76.9 ± 16.6	94.1 ± 24.5	0.040	69.5 ± 16.3	65.4 ± 17.6	0.321	0.356	0.012
FEV_1_, L	2.7 ± 0.9	3.5 ± 1.0	0.010	2.5 ± 0.6	2.4 ± 0.6	0.601	0.576	0.013
FEF_75_, L/s	4.9 ± 1.9	6.6 ± 1.8	0.011	4.9 ± 2.0	5.9 ± 1.8	0.099	0.967	0.434
FEF_50_, L/s	3.6 ± 1.5	4.3 ± 1.6	0.092	3.2 ± 1.1	3.5 (0.9)^†^	0.944	0.606	0.354
FEF_25_, L/s	1.7 ± 0.9	1.9 ± 1.1	0.581	1.7 ± 0.7	1.5 ± 1.0	0.470	0.891	0.417
PEF, L/s	5.3 ± 1.6	7.1 ± 2.0	0.010	5.1 ± 2.2	6.0 ± 2.2	0.204	0.834	0.289
MVV, L	97.8 ± 40.3	117.1 ± 30.3	0.017	96.7 ± 34.0	104.0 ± 29.5	0.204	0.952	0.364

*EAW* exoskeleton-assisted walking, *FVC* forced vital capacity, *FEV*_*1*_ forced expiratory volume in 1 s, *FEF* forced expiratory flow, *PEF* peak expiratory flow, *MVV* maximal voluntary ventilation, *SD* standard deviation, *IQR* interquartile range

^†^Report the median (IQR)

Participants who treated with EAW training had statistical improvements from pre- to post-intervention in mean change in predicted FVC% (Δ = 17.2%; t = 2.445; p = 0.040), FEV_1_ (Δ = 0.8 L; t = 3.359; p = 0.010), FEF_75_ (Δ = 1.7 L/s; t = 3.268; p = 0.011), PEF (Δ = 1.8 L/s; t = 3.381; p = 0.010), and MVV (Δ = 19.3 L; t = 3.274; p = 0.017). Howbeit, there was no statistically significant differences in FVC (Δ = 0.7 L; t = 2.275; p = 0.052), FEF_50_ (Δ = 0.6 L/s; t = 1.917; p = 0.092), and FEF_25_ (Δ = 0.2 L/s; t = 0.575; p = 0.581). Nonetheless, there was no evidence of statistical improvements from pre- to post-intervention for individuals who received conventional training in FVC (Δ = − 0.2 L; t = − 1.146; p = 0.285), predicted FVC% (Δ = − 4.1% L; t = − 1.057; p = 0.321), FEV_1_ (Δ = − 0.1 L; t = − 0.544; p = 0.601), FEF_75_ (Δ = 1.0 L/s; t = 1.865; p = 0.099), FEF_50_ (Z = − 0.70; p = 0.944), FEF_25_ (Δ = − 0.2 L/s; t = − 0.758; p = 0.470), PEF (Δ = 0.9 L/s; t = 1.383; p = 0.204), and MVV (Δ = 7.3 L; t = 1.364; p = 0.204).

### Secondary outcomes

Of the 10 participants who completed the final 6MWT, 2 were in the conventional group. All participants in EAW group completed it while wearing the exoskeleton in door. A summary of secondary outcomes is provided in Table 3. The outcomes of distance recording as medians (IQR) were 17.3 (11.9) meters and 0 (16.0) meter for EAW and conventional group, respectively. Nonetheless, EAW training produced no statistical improvements in distance (Z = 1.756; p = 0.079) and SpO_2_ (− 2%; t = 2.032; p = 0.059) than conventional group. There were differences in HR (Z = 2.311; p = 0.021), and RPE (Z = 2.330; t = 0.020) between groups. Moreover, participants who treated with EAW training had differences from pre- to post- intervention in HR (Δ = 20.2; t = 4.293; p = 0.003), and PRE (Z = 2.558; p = 0.011). Howbeit, distance (Z = 1.400; p = 0.161) and SpO_2_ (Δ = 3.8%; t = − 1.474; p = 0.179) showed no differences. For conventional group, no statistical differences were reported for distance (Z = 1.342; t = 0.180), HR (Z = 0.351 = 0.725), SpO_2_ (Δ = 1.7%; t = 2.236; p = 0.056) and RPE (Z = 0.447; p = 0.655). The outcomes of correlation between the distance of 6MWT and evert item of PFT are shown in Additional file 1: Appendix 1. The distance showed a significant positive correlation with FEV_1_ (Pearson correlation coefficient 0.741, p = 0.022) and FEF_75_ (Pearson correlation coefficient 0.688, p = 0.040).

**Table 3:** Outcomes of 6-min walk test (6MWT) and lower extremity motor scores (LEMS)

Characteristic, median (IQR)	EAW group (n = 9)			Conventional group (n = 9)			p-values between groups	
	Pre	Post	p-value in group	Pre	Post	p-value in group	Pre	Post
Distance	0 (0)	17.3 (11.9)	0.161	0 (0)	0 (16.0)	0.180	0.999	0.079
Herat rate	86.8 ± 10.3^‡^	107 ± 19.6^‡^	0.003	84.3 ± 7.2^‡^	87 (12.5)	0.725	0.569	0.021
RPE	0 (0)	2.0 (2.0)	0.011	0(0)	0 (1.5)	0.655	0.999	0.020
SpO_2_^‡^	95.7% ± 1.2%	95.1% ± 1.5%	0.179	95.4% ± 2.1%	97.1% ± 2.5%	0.056	0.785	0.059
LEMS	0 (1.5)	1.0 (16)	0.180	0 (0)	0 (11.0)	0.068	0.297	0.777

^‡^Report the mean ± standard deviation, *RPE* rate of perceived exertion, *SpO*_*2*_ peripheral oxygen saturation, *IQR* interquartile range

For LEMS, there was no statistical difference between two groups (Z = 0.283; p = 0.777). Additionally, neither groups showed improvement in LEMS (Z = 1.342; p = 0.180 in EAW group; Z = 1.826; p = 0.068 in conventional group). More detailed data are provided in Additional file 2.

## Discussion

This study focused on the changes of PFT and 6MWT using EAW and conventional trainings in individuals with T4 to L1 SCI. Robotic exoskeleton has been explored and applied in rehabilitation treatment after SCI. At present, the robotic exoskeleton has demonstrated some advantages in assisting walking and improving physical functions [34, 35]. We found that EAW trainings provided statistically significant improvement in FVC, predicted FVC% and FEV_1_ compared with conventional trainings. Nonetheless, there were no differences in FEF, PEF, and MVV between two groups. Moreover, EAW trainings offered statistically different results of PFT than the beginning, except FVC, FEF_50_ and FEF_25_.

### Exoskeleton-assisted walking training enhanced the pulmonary function parameters among individuals with spinal cord injury

Some previous studies [23–25, 35] have shown that robotic exoskeletons provide improvement in VO_2 peak_ among the incomplete SCI individuals during gait training. We reported changes in PF parameters among complete and incomplete SCI individuals. In the present study, it was reported better results in FVC, predicted FVC% and FEV_1_ compared with the conventional trainings that were widely used in SCI rehabilitation program. There were statistical differences in post-predicted FVC%, FEV_1_, FEF_75_, PEF and MVV after EAW compared with pre-outcomes. The improvements in PF parameters might reflect the increasing of respiratory muscle strength and pulmonary ventilation. Alamro et al. [36] has demonstrated that overground walking by exoskeleton elicits greater activation of trunk muscles compared with treadmill walking, even after controlling for the use of hand-held assistive devices. Moreover, Guan et al. [37] also reported EAW had advantages over conventional gait orthosis on recruiting muscles. Hence, the underlying mechanisms of how EAW improved the PF parameters might be the potential to recruit trunk muscles. The improvements of trunk muscles in SCI individuals resulted in better pulmonary function [38, 39].

The increasing or maintenance of PF parameters has crucial meanings for many patients with SCI. Hart et al. [40] has reported that lower FEV_1_ and FVC associate with higher inflammatory factors that reflect systemic inflammation. Hence, better results of FEV_1_ and FVC are beneficial to reduce systemic inflammation and manifested better functions of principal bronchus. Although there was no difference in pre- and post- FVC in 2 groups, we reported a higher mean value after EAW training. At least, both trainings helped to maintain the FVC, and EAW might improve the FVC. Moreover, the minimal clinically important difference for predicted FVC% is 2–6% by distribution-based method [41]. The mean changes of difference between groups and from pre- to post-intervention in EAW group were beyond the threshold which were 28.7% and 17.2%, respectively. Additionally, the minimal important difference of FEV_1_ is 0.1 L according to previous study by the anchor-based method [42], which is smaller than our results (1.1 L between groups; 0.8 L in EAW group). Therefore, EAW training has potential benefits of both clinical and statistical meanings for PF parameters.

As for the conventional trainings which are recommended and wildly used for many years [43–45], this study only demonstrated the capability to keep PFT among individuals with SCI. Nevertheless, the changes could be secondary to natural recovery since all participants in conventional group were in acute stage. In spite of that, the differences between groups still proved the advantages of EAW. Nonetheless, Akkurt et al. [46] reported that upper extremity aerobic exercise aimed at 50–70% VO_2 max_ improves exercise capacity among individuals with C7 to L5 SCI. In this study, the HR values during the trainings reached a moderate-intensity level based on the ACSM guidelines for exercise testing and prescription [47]. The inconsistent results may be caused by different intensities and ranges of included injury level.

### Robotic exoskeleton improved walking ability

Robotic exoskeleton was achievable to enhance the walking capacity. Furthermore, no improvement in LEMS also manifested that EAW effect on walking capacity, not no motor function or lower limb muscle performance. Our result of 6MWT was similar to McIntosh et al. [48] and Sale et al. [49]. All participants were able to perform walking while wearing the exoskeleton. Nevertheless, only two participants in conventional group had knee-ankle–foot orthoses and completed the 6MWT. Others were limited to the access of brace in this study.

For individuals with SCI, the results of 6MWT might not report the cardiopulmonary endurance completely. In this study, the distance was partially related to PFT. There was no statistically significant collection between the distance and most item of PFT. This might be influenced by the primary disease, different assisted devices and the using duration. Longer using duration may result in longer distance. Benson et al. [50] found the minimal distance of 6MWT after 10-week trainings was 91 m which was more than 5 times than our average distance. Additionally, we found that individuals with lower injury level (T11–L1) had better improvements (17.7 ± 4.3 m) than others (14.3 ± 7.7 m) in distance which did not provide statistical difference (p = 0.481). This was partly confirmed by Louie et al. [51] and Guanziroli et al. [52] who reported walking speed and performances were significantly associated with injury level. Additionally, we did not report any adverse event, even more than half of the individuals in EAW group was acute inpatient.

### Study limitations

This study was limited to the small sample size and number of training session, although our feasibility study has proved this training period realized the application of EAW and was most achievable in inpatient rehabilitation in the health care system. The results in this study may not be generalized to larger population. It is necessary to explore the sample size and future investigation on the effectiveness of EAW. Although we tried to avoid the detection bias, it was inevitable during the 6MWT. A majority of subjects from the EAW group was AIS A level that may influence the results of 6MWT, although there was no difference of LEMS at the baseline. Moreover, further study needs to have control groups composed of healthy participants with EAW training or conventional training, and compare with individuals with SCI.

Furthermore, the intensity calculated by the HR was rough and imprecise in some degree. The predict HR_max_ by the formula: HR_max_ = 220—age might be inaccurate. HR_max_ is individual and related to sex and physical activity status [53]. In this study, the range of age is 40 and the used equation underestimates the HR_max_ in older adults [28]. Moreover, the validity of equation has not been established in a study sample that included an adequate number of individuals with SCI. This might have resulted in misestimation of the training intensity, which in turn, result in comparison of PFT. Hence, cardiopulmonary exercise test is needed possibly for accurate cardiopulmonary performance parameters. Last, gait parameters should be recorded and compared while walking without the exoskeleton for participants who had the capacity for walking.

## Conclusions

The study successfully manifested that EAW training has the potential to improve performance in PF parameters among individuals with SCI, especially in ventilation. Additionally, robotic exoskeleton is a device that can assist individuals with SCI to stand up and walk. The results suggested that the effectiveness of EAW might be similar, or better than conventional training. Further research is essential to confirm these results in a larger sample size and better study protocols. The data in this study will be helpful for further efforts towards improving the design and clinical application of EAW.

## Supplementary Information

**Additional file 1.** Outcomes of correlation between the distance of 6-minute walk test (6MWT) and every item of pulmonary function test (PFT).

**Additional file 2.** Individual-participant data.

## Data Availability

The dataset used in the study is available from the corresponding author on reasonable request.

## Abbreviations

SCI: Spinal cord injury
PF: Pulmonary function
EAW: Exoskeleton-assisted walking
FVC: Forced vital capacity
FEV_1_: Forced expiratory volume in 1 s
MVV: Maximal voluntary ventilation
ASIA: American spinal injuries association impairment scale
AIDER: AssItive DEvice for paRalyzed patient
HR: Heart rate
PFT: Pulmonary function test
FEF: Forced expiratory flow
PEF: Peak expiratory flow
SpO_2_: Peripheral oxygen saturation
RPE: Rate of perceived exertion
6MWT: 6-Minute walk test
LEMS: Lower extremity motor score
CI: Confidence intervals
IQR: Inter quartile range
