# Supplementary material for: Exoskeleton-assisted walking improves pulmonary function and walking parameters among individuals with spinal cord injury: a randomized controlled pilot study
Source: J Neuroeng Rehabil. 2021 May 24;18:86. doi: 10.1186/s12984-021-00880-w (PMC8146689; doi:10.1186/s12984-021-00880-w)
Supplement: Supplementary file 1 — Additional file 1. Outcomes of correlation between the distance of 6-minute walk test (6MWT) and every item of pulmonary function test (PFT). [file 12984_2021_880_MOESM1_ESM.docx]

**Appendix 1.**

| Characteristic | Pearson correlation coefficient | p-value |
| --- | --- | --- |
| Distance-FVC | 0.821 | 0.063 |
| Distance-FEV_1_ | 0.741 | 0.022 |
| Distance-FEF_75_ | 0.688 | 0.040 |
| Distance-FEF_50_ | 0.371 | 0.326 |
| Distance-FEF_25_ | 0.310 | 0.417 |
| Distance-PEF | 0.641 | 0.063 |
| Distance-MVV | 0.641 | 0.063 |
